# An ensemble deep learning model for risk stratification of invasive lung adenocarcinoma using thin-slice CT

**DOI:** 10.1038/s41746-023-00866-z

**Published:** 2023-07-05

**Authors:** Jing Zhou, Bin Hu, Wei Feng, Zhang Zhang, Xiaotong Fu, Handie Shao, Hansheng Wang, Longyu Jin, Siyuan Ai, Ying Ji

**Affiliations:** 1 Center for Applied Statistics, School of Statistics, Renmin University of China, Beijing, China; 2 Department of Thoracic Surgery, Beijing Institute of Respiratory Medicine and Beijing Chao-Yang Hospital, Capital Medical University, Beijing, China; 3 Department of Cardiothoracic Surgery, The Third Xiangya Hospital of Central South University, Changsha, China; 4 Department of Thoracic Surgery, Changsha Central Hospital, Changsha, China; 5 Guanghua School of Management, Peking University, Beijing, China; 6 Department of Thoracic Surgery, Beijing LIANGXIANG Hospital, Beijing, China

**Keywords:** Cancer imaging, Non-small-cell lung cancer

## Abstract

Lung cancer screening using computed tomography (CT) has increased the detection rate of small pulmonary nodules and early-stage lung adenocarcinoma. It would be clinically meaningful to accurate assessment of the nodule histology by CT scans with advanced deep learning algorithms. However, recent studies mainly focus on predicting benign and malignant nodules, lacking of model for the risk stratification of invasive adenocarcinoma. We propose an ensemble multi-view 3D convolutional neural network (EMV-3D-CNN) model to study the risk stratification of lung adenocarcinoma. We include 1075 lung nodules (≤30 mm and ≥4 mm) with preoperative thin-section CT scans and definite pathology confirmed by surgery. Our model achieves a state-of-art performance of 91.3% and 92.9% AUC for diagnosis of benign/malignant and pre-invasive/invasive nodules, respectively. Importantly, our model outperforms senior doctors in risk stratification of invasive adenocarcinoma with 77.6% accuracy [i.e., Grades 1, 2, 3]). It provides detailed predictive histological information for the surgical management of pulmonary nodules. Finally, for user-friendly access, the proposed model is implemented as a web-based system (https://seeyourlung.com.cn).

## Introduction

Lung cancer has always been among the most frequently diagnosed cancers threatening people’s health worldwide. In 2020, the detection rate was approximately 11.4% of all cancer diagnoses, ranking in the top place^1^. Moreover, it is also the leading cause of cancer-related mortality, accounting for approximately 18% of total cancer-related deaths^1^. In China, the incidence of lung cancer and cancer-related mortality in 2020 ranked in the first place among all cancers, with an overall 5-year survival rate of approximately 30%^2^. In recent decades, with the popularization of low-dose computed tomography (LDCT) in lung cancer screening, more early-stage lung cancers have been detected^3^. The critical role of LDCT screening in reducing lung cancer-related mortality has been confirmed by evidence-based medicine^4,5^.

According to the classification of the International Association for the Study of Lung Cancer (IASLC), lung adenocarcinoma can be divided into two broad categories: pre-invasive adenocarcinoma (Pre-IA) and invasive adenocarcinoma (IAC)^6^. Pre-IA consists of atypical adenomatous hyperplasia (AAH), adenocarcinoma in situ (AIS), and minimally invasive adenocarcinoma (MIA). Besides, according to the latest IASLC grading system, predominant and high-grade patterns are practical and prognostic for invasive lung adenocarcinoma^7^. This new grading system divides IAC into three risk grades and shows satisfactory survivability hierarchical evaluation. It is meaningful to predict the pathological grades of IAC by CT images since it helps select the most suitable surgical approach (lobectomy, wedge resection, or segmentectomy) before performing a medical operation. However, the current lung cancer screening results based on chest CT may result in different interpretations by different surgeons, posing serious challenges to clinical work. These clinical challenges motivate us to develop an automated diagnosis system. Such a system should not only quickly and accurately detect and classify benign and malignant nodules but also the pre-invasive and invasive types, as well as the IAC pathological grades.

To detect and diagnose pulmonary nodules, some studies have used extracted imaging features to predict the pathological type^8–12^. For example, researchers developed radiomic models to extract thousands of tumor-related features to quantify the image features of lung tumors, such as morphological, texture, boundary, and intensity features^13–17^. Wu et al.^18^ demonstrated that separating ground-glass and solid CT radiomic features of part-solid nodules is useful in diagnosing the invasiveness of lung adenocarcinoma. Wang et al.^19^ combined a radiomic method and frozen sections to predict the final classification of peripheral lung adenocarcinoma manifesting as ground-glass nodules. Although the radiomic-based method has been proven to be effective in predicting the tumor pathological type, it is limited by the number of extracted features. This is because these extracted features highly rely on human engineering and the radiologist’s subjective intervention, which may cause an unnecessary subjective bias.

Recently, with the rapid development of deep learning technologies, features that can be automatically learned using convolutional neural network (CNN) models have been verified as a great supplementary to hand-crafted features extracted using radiomic models^20,21^. CNN-based methods have been successful in many lung cancer-related tasks, such as tumor segmentation^22–26^, benign and malignant classification^27–31^, and tumor prognosis^27,32–34^. For most of the benign-malignant discrimination tasks, researchers usually validate their models by either using screen-detected nodules^35^ or incidentally detected nodules^36,37^, which were with low incident rate of lung cancer. That means, those proposed models may be more suitable for screening populations with less suspicious nodules. In addition to lung cancer screening research, some scholars are dedicated to distinguishing invasive lesions in malignant nodules^38–42^. For example, Gong et al.^43^ developed a deep neural network model to diagnose ground-glass nodules and classify Pre-IA and IAC. However, the results of their model on two external validation sets were unstable (Their model on validation 1 only yielded an AUC value of 0.76, while the AUC value for validation 2 was 0.96). Besides, most previous studies only focused on a certain type of nodules in the inclusion criteria. Some researchers focused on solid or semisolid nodules^35–37^, while others focus on ground-glass nodule or mixed grounded glass nodule^38,41,43^. This may limit the real-world application of AI models.

In summary, all the aforementioned studies focused on classifying benign and malignant pulmonary nodules or Pre-IA and IAC. To the best of our knowledge, there is currently no research on the pathological grading of IAC despite its importance for thoracic surgeons to select the most effective surgical treatment^44^. For pre-invasive lesions, with their excellent prognosis, sublobar resection is usually sufficient. Nevertheless, the histological subtypes can significantly affect outcomes in IAC, which could aid in selecting the optimal resection procedure^45^. To this end, it is of great practical importance to correctly determine the IAC risk stratification levels by screening CT images.

**Fig. 1:**
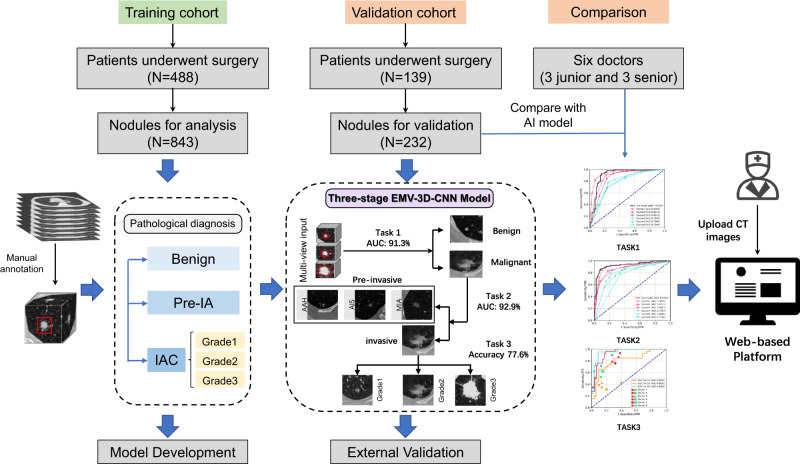
Study design and workflow of the study. The training cohort is used to develop our model. The validation cohort is used to evaluate the model performance. A comparison study is conducted between our model and two groups of six radiologists. Finally, we develop a user-friendly web-based platform for doctors.

To overcome the limitations of previous literature, we develop a three-stage ensemble multi-view 3D convolutional neural network (EMV-3D-CNN) model to diagnose benign and malignant lung tumors (Task 1), classify Pre-IA and IAC (Task 2), and further identify the risk stratification level (i.e., Grades 1, 2, and 3) of IAC (Task 3; see the Methods section for the detailed definitions of the three grades). Here, we adopt a heterogeneous ensemble (HEE) strategy, which is a general ensemble strategy that require multiple models trained on the sane dataset^46^. Figure 1 shows an overview of our study. To broaden the applicability of our model, we include various types of pulmonary nodules, such as pure-solid, part-solid, pure ground-glass and heterogeneous ground-glass nodules. Our approach consists of three key tasks. For each task, we first independently train three 3D CNN models (e.g., 3D Inception, 3D VGG, and 3D ResNet) on the training set with the pathological types as targeted labels. With the best trained model, each 3D CNN model can give a predicted probability of each category for each pulmonary nodule in the validation dataset. We then calculate an averaged probability of each category for each nodule. For Task 1 and Task 2, a cut-off value determined using the Youden index is adopted to obtain the final predicted label. For Task 3, the category corresponding to the maximum averaged prediction probability is used as the final predicted label. To further evaluate the model performance, a retrospective reader study with six certified doctors is conducted by comparing the performance of the six doctors and the proposed model concerning lung adenocarcinoma risk stratification. Finally, for user-friendly access, the proposed model is also implement as a web-based system (https://seeyourlung.com.cn). By uploading one full original CT image in the DICOM format for a patient, our algorithm can give the probability of malignancy of pulmonary nodules by specifying the center location of the target lung nodule.

Compared to previous studies, our contributions can be summarized as follows. First, we develop an AI tool that more likely works for suspicious nodules that will undergo a surgery. Second, by including various types of pulmonary nodules, the proposed AI tool is more widely applicable in the real world. Finally, we not only fill the gap of automatic diagnosis for identifying the risk stratification levels of IAC but also provide an artificial intelligence (AI)-based diagnosis platform for surgeons to determine the most effective surgical treatment before performing a medical operation.

## Results

### Datasets

A total of 1,075 pulmonary nodules with confirmed pathological results were collected from 627 subjects (252 men, 375 women, mean age of 58.1 ± 11.6 years) in three centers (from Jan 2016 to Dec 2021) to develop and validate the proposed EMV-3D-CNN model. The training and validation cohorts included 488 (Beijing Chao-Yang Hospital) and 139 subjects (The Third Xiangya Hospital of Central South University: 89, Changsha Central Hospital: 50), respectively. It should be noted that one sample unit in our study represents a specific pulmonary nodule. Accordingly, the training and validation sets included 843 and 232 pulmonary nodules, respectively. All the patients included in this study received a thin-slice CT scan with slice thickness ranging from 0.5 mm to 1.5 mm, with an average of 0.625 mm. No significant differences were observed between each center (*p* > 0.05). Main inclusion criteria were nodules (≤30 mm and ≥4 mm) with definite pathology confirmed by surgery. Supplementary Note 1 shows the detailed inclusion/exclusion criteria for the registered patients in this study. Table 1 summarizes the demographics and clinical characteristics of the patients in the training and validation cohorts, and Supplementary Table 1 lists the CT scan characteristics for the three centers that participated in this study. To identify the pathological type of each pulmonary, we treated each pulmonary nodule as an independent observation, and its position was labeled by reading the CT image before the surgery. To locate the position, the center point of each nodule on the CT scan was recorded using *X, Y*, and *Z* coordinates, along with the maximum diameter of each tumor in millimeters (mm). Details of the labeling procedure are described in Supplementary Note 2.

**Table 1:** Demographic and clinical characteristics of patients in the training and validation datasets.

		Training dataset (*n* = 843)	Validation dataset (*n* = 232)
Age Mean (SD)		58.4(±11.5)	57.7(±11.7)
Gender	Male	198(40.6%)	54(38.8%)
	Female	290(59.4%)	85(61.2%)
Pathological type	Benign	216(25.6%)	78(33.6%)
	Pre-IA	288(34.2%)	78(33.6%)
	IAC (Grade1)	124(14.7%)	29(12.5%)
	IAC (Grade2)	116(13.8%)	26(11.2%)
	IAC (Grade 3)	99(11.7%)	21(9.1%)
Location	LUL	200(23.7%)	49(21.1%)
	LLL	140(16.6%)	32(13.8%)
	RUL	268(31.8%)	88(38.0%)
	RML	78(9.3%)	15(6.5%)
	RLL	157(18.6%)	48(20.7%)
Nodule morphology	pGGN	287(34.0%)	63(27.2%)
	Heterogeneous ground-glass nodule	41(4.9%)	20(8.6%)
	Part-solid nodule	182(21.6%)	55(23.7%)
	Pure solid nodule	333(39.5%)	94(40.5%)
Diameter (mm)	≤10 mm	429(50.9%)	122(52.6%)
	10–20 mm	280(33.2%)	72(31.0%)
	20–30 mm	134(15.9%)	38(16.4%)
Average diameter (mm) Mean (SD)	Benign Malignant	8.6( ± 7.6) 14.3( ± 8.8)	8.9( ± 7.3) 14.2( ± 8.1)

*n* is the number of nodules, *IAC* invasive adenocarcinoma, *RUL* right upper lobe, *RML* right middle lobe, *RLL* right lower lobe, *LUL* left upper lobe, *LLL* left lower lobe, *pGGN* pure ground-glass nodule.

### Model performance for the three tasks

We trained and evaluated the EMV-3D-CNN model on the training and validation sets for the three tasks, then compared its performance with that of radiologists. Six doctors from Beijing Chao-Yang Hospital and The Third Xiangya Hospital of Central South University, who had 2–15 years of clinical experience with an average of 8 years, independently graded the CT images in the validation set to obtain the classification results of a specific pulmonary nodule. They comprised two groups: a senior group (Doctors 1–3) with an average experience of 13 years (reading an average of 3500 chest CT scans per annum) and a junior group (Doctors 4–6) with an average experience of 3 years (reading an average of 2400 chest CT scans per annum). More details for this observational study are provided in the Methods section.

**Fig. 2:**
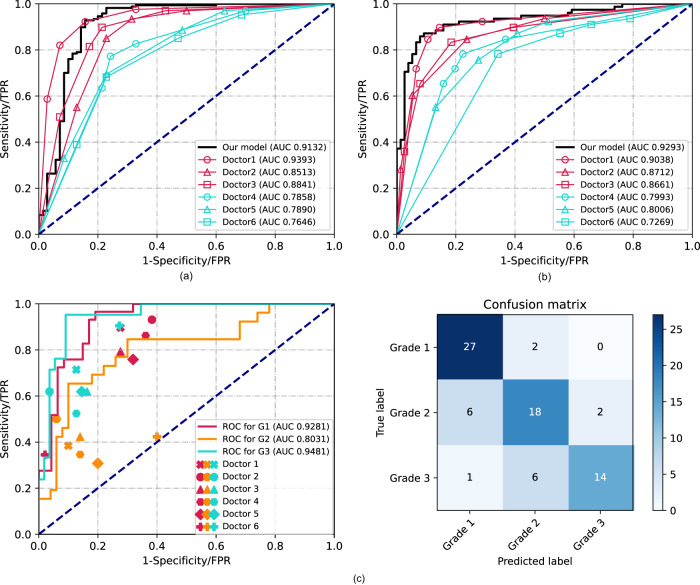
Comparisons of ROC curves and AUC values generated by the proposed EMV-3D-CNN model and the six doctors. **a** The Task 1 ROC curves for our model and the six radiologists. **b** The Task 2 ROC curves for our model and the six radiologists. **c** For the multiclass problem of Task 3, we conduct a pairwise comparison (one class *vs*. all other classes) and then plot three standard ROC curves. For the radiologists’ study, red, yellow, and blue indicate the performance of identifying the nodule risk for Grades 1, 2, and 3, respectively.

We first evaluated the proposed model for Task 1, identifying benign and malignant pulmonary nodules. Figure 2a shows the receiver operating characteristic (ROC) curves of the proposed model and the six doctors by testing on the validation set. The proposed EMV-3D-CNN model achieved an area under the curve (AUC) of 91.3% (95% confidence interval: 85.6–96.2%). We defined an optimal cut-off value of 0.747 for the EMV-3D-CNN model according to the Youden index. The results showed that the proposed model achieved a sensitivity, specificity, positive predictive value (PPV), negative predictive value (NPV), and accuracy of 0.928, 0.843, 0.934, 0.831, and 0.903, respectively. With a PPV of 0.934, the model achieved a sensitivity of 0.928, which indicates that the proposed model can identify 92.8% of the malignant cases by only using a thin-slice chest CT scan, when allowing 6.6% of the positive predictions as false. In addition, our model achieved a specificity of 0.843, which suggests the probability of diagnosing a benign nodule as malignant is only 15.7%. Table 2 lists and summarizes the performance evaluation metrics (see Methods, Statistical analysis) for both the EMV-3D-CNN model and the six doctors by testing on the validation set. For the radiologist performance, the senior (i.e., Doctors 1–3) and junior groups (i.e., Doctors 4–6) yielded average AUC values of 89.16% and 77.98%, respectively. The more experienced the radiologists were, the better the AUC values achieved in the classification of Task 1. This verifies that the EMV-3D-CNN model achieved equivalent or slightly higher performance compared with senior doctors, and much higher performance compared with junior doctors.

**Table 2:** Performance comparisons of the proposed EMV-3D-CNN model and the six radiologists on the validation dataset for Tasks 1 and 2.

Evaluation Index	Task 1							Task 2						
	Model	D1	D2	D3	D4	D5	D6	Model	D1	D2	D3	D4	D5	D6
AUC (%)	91.3	93.9	85.1	88.4	78.6	78.9	76.5	92.9	90.4	87.1	86.6	79.9	80.1	72.7
Accuracy (%)	90.3	89.9	86.1	86.5	78.1	77.6	75.5	89.0	87.7	80.5	82.5	77.9	73.4	63.0
Sensitivity (%)	92.8	92.2	93.4	89.8	82.6	88.6	85.0	85.9	89.7	84.6	83.3	78.2	87.2	91.0
Specificity (%)	84.3	84.3	68.6	78.6	67.1	51.4	52.9	92.1	85.5	76.3	81.6	77.6	59.2	34.2
PPV (%)	93.4	93.3	87.6	90.9	85.7	81.3	81.1	91.8	86.4	78.6	82.3	78.2	68.7	58.7
NPV (%)	83.1	81.9	81.4	76.4	61.8	65.5	59.7	86.4	89.0	82.9	82.7	77.6	81.8	78.7
F1 (%)	93.1	92.8	90.4	90.4	84.1	84.8	83.0	88.7	88.1	81.5	82.8	78.2	76.8	71.4

*PPV* positive predictive value, *NPV* negative predictive value, *D* Doctor.

Second, we evaluated the proposed model in terms of distinguishing Pre-IA from IAC (i.e., Task 2). Figure 2b shows the ROC curves of the proposed model and the six doctors by testing on the validation set. The EMV-3D-CNN model achieved an AUC of 92.9% (95% confidence interval: 88.2–96.6%). Similar to Task 1, we defined an optimal cut-off value of 0.46 for the EMV-3D-CNN model according to the Youden index. Consequently, the proposed model’s sensitivity, specificity, PPV, NPV, and accuracy were 0.859, 0.921, 0.918, 0.864, and 0.890, respectively. This suggests that the proposed model can identify 85.9% of the invasive cases when controlling the false positive rate to be as low as 7.9%. For the radiologists’ performance, the senior (Doctors 1–3) and junior groups (Doctors 4–6) yielded average AUC values of 88.04% and 77.56%, respectively. Table 2 lists and summarizes the performance evaluation metrics for both the EMV-3D-CNN model and the six doctors by testing on the validation set. The evaluation metrics revealed that the EMV-3D-CNN model achieved higher performance than the radiologists in distinguishing invasive nodules from pre-invasive ones.

Lastly, we evaluated the EMV-3D-CNN model for identifying the pathological risk stratification of IAC (i.e., Task 3). Since Task 3 is a multiclass classification problem, the standard ROC-related evaluation metrics could not be directly applied. To this end, following Landgrebe et al.^47^, we carried out a pairwise comparison strategy (one class vs. all the other classes), and for each pair, we plotted a standard ROC curve. Specifically, for Task 3, we plotted three standard ROC curves indicating the classification performance for each grade vs. the other two grades. For the radiologists’ observational study, we could only give each doctor one data point specifying the false positive rate and true positive rate for each task. The left panel of Fig. 2c presents the multiclass ROC curves. From the figure, the AUC value of Grade 3 was 94.8%, followed by Grade 1 (AUC: 92.8%), and Grade 2 was the most difficult pathological grade to be identified, with an AUC value of only 80.3%. Furthermore, the confusion matrix in the right panel of Fig. 2c supports the above findings. From the confusion matrix, among pulmonary nodules with the true pathological type of Grade 2, 23.1% were incorrectly predicted as Grade 1. This number increased to 28.6% when the true pathological type was Grade 3, and the result was incorrectly predicted as Grade 2. However, the misclassification rate for Grade 1 was only 6.9%, which is low. For more detailed confusion matrix results for the six doctors, we refer to Supplementary Fig. 2. Table 3 lists the model accuracy results. It can be seen that the proposed EMV-3D-CNN model achieved an accuracy of 77.6%, which was much higher than those of the senior (average accuracy of 66.23%) and junior groups (average accuracy of 56.14%). These results verified that the EMV-3D-CNN model achieved much higher performance in pulmonary IAC risk stratification than doctors.

**Table 3:** Performance comparisons of the proposed EMV-3D-CNN model and the six radiologists on the validation dataset for Task 3.

Overall accuracy (%)	Task 3																				
	Model			D1			D2			D3			D4			D5			D6		
	77.6			67.1			69.7			61.8			59.2			56.6			52.6		
	G1	G2	G3	G1	G2	G3	G1	G2	G3	G1	G2	G3	G1	G2	G3	G1	G2	G3	G1	G2	G3
Accuracy (%)	88.2	78.9	88.2	78.9	72.4	82.9	73.7	78.9	86.8	75.0	71.1	77.6	72.4	68.4	77.6	71.1	63.2	78.9	73.7	53.9	77.6
Sensitivity (%)	93.1	69.2	66.7	89.7	38.5	71.4	93.1	50.0	61.9	79.3	42.3	61.9	86.2	34.6	52.4	75.9	30.8	61.9	34.5	42.3	90.5
Specificity (%)	85.1	84.0	96.4	72.3	90.0	87.3	61.7	94.0	96.4	72.3	86.0	83.6	63.8	86.0	87.3	68.1	80.0	85.5	97.9	60.0	72.7
PPV (%)	79.4	69.2	87.5	66.7	66.7	68.2	60.0	81.3	86.7	63.9	61.1	59.1	59.5	56.3	61.1	59.5	44.4	61.9	90.9	35.5	55.9
NPV (%)	95.2	84.0	88.3	91.9	91.9	88.9	93.5	78.3	86.9	85.0	74.1	85.2	88.2	71.7	82.8	82.1	69.0	85.5	70.8	66.7	95.2
F1 (%)	85.7	69.2	75.7	76.5	76.5	69.8	73.0	61.9	72.2	70.1	50.0	60.5	70.4	42.9	56.4	66.7	36.4	61.9	50.0	38.6	69.1

*PPV* positive predictive value, *NPV* negative predictive value, *D* Doctor, *G* Grade.

**Fig. 3:**
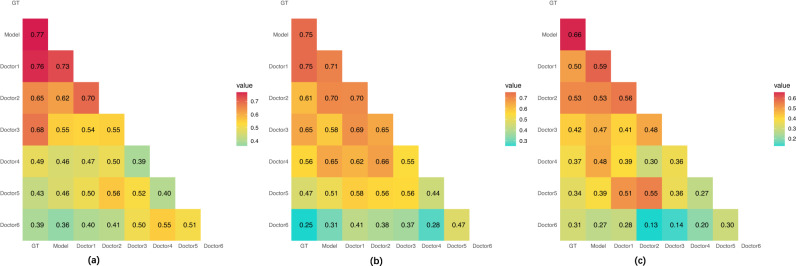
Cohen’s kappa values for the EMV-3D-CNN model and six radiologists. **a** Task1, **b** Task2, **c** Task3. GT stands for the pathological ground truth. The kappa values are interpreted as follows: < 0.2: poor consistency; 0.21–0.4: fair consistency; 0.41–0.6: moderate consistency; 0.61–0.8: slightly strong consistency; 0.81–1.0: strong consistency.

For all three tasks, we calculated Cohen’s kappa values to measure the interrater reliability of the EMV-3D-CNN model and the six doctors compared with the ground truth (GT) of the histopathological results. Figure 3 presents the results in the form of a heat map. For Tasks 1 and 2, the binary classification results for the six doctors were generated by categorizing the prediction score of 3 into the high-risk group (i.e., malignant group or invasive group). Compared with the GT of nodules, both the EMV-3D-CNN model and the senior group showed relatively high agreement, and this consistency decreased with the decrease in the radiologist’s experience. Lastly, to assess the benefit of adopting an ensemble strategy, we also provide the performance for each individual models (i.e., 3D Inception, 3D VGG, and 3D ResNet) in the EMV-3D-CNN model for all the three tasks. The detailed performance results can be found in Supplementary Tables 2–3 and Supplementary Fig. 3. For example, from the result of task 1 in Supplementary Table 2, the AUC values of the three individual models are not as good as that of the integrated model. Similar patterns can also be found in task 2. Additionally, the improvement (i.e., accuracy value) of the ensemble model for task 3 is much larger than each of the individual model. All of these evidences have shown that applying individual model is not good enough. Therefore, we propose this ensemble model.

### Web-based platform

**Fig. 4:**
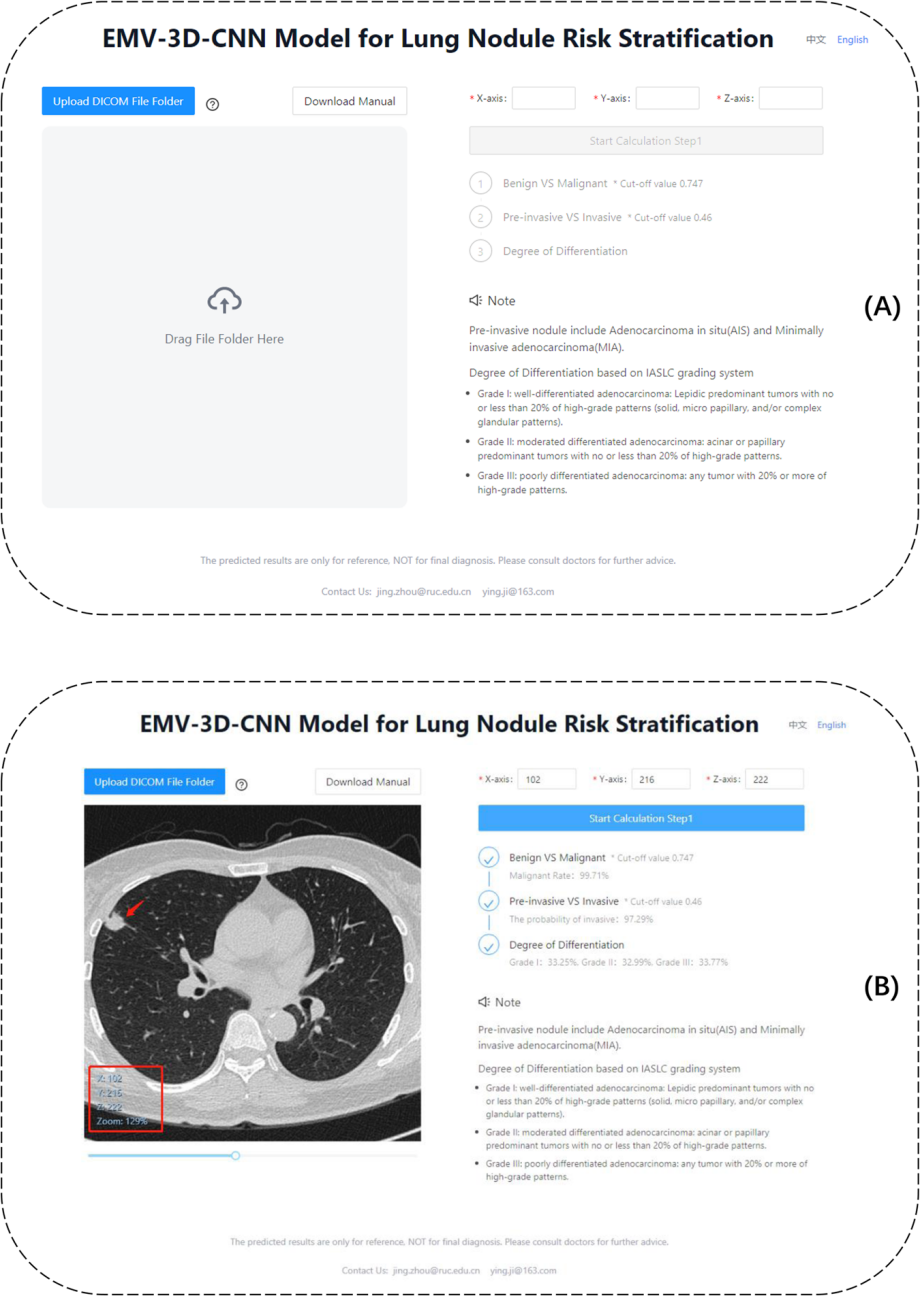
Snapshot of the web-based platform. Panel (**A**) is a snapshot of the website before users upload a series of CT images. Panel (**B**) is a snapshot of the website after users upload a series of CT images. The left side of the page displays the CT images. Doctors can find the lung nodules by sliding the scroll bar below. Once they find the nodule, its corresponding *X, Y*, and *Z* coordinates can be recorded manually. On the right side, our algorithm can give the predictive results of Tasks 1–3 by clicking the start calculation button. For a detailed user guideline, doctors can either go to the website (https://seeyourlung.com.cn) to download the manual or refer to the Supplementary Note 3.

For many clinicians, deep learning (or machine learning) methods represent a black box that is difficult to use and interpret. Thus, one of the ultimate goals for clinicians is to use easily interpretable deep learning-based models in practice. For example, in our case, once doctors upload a series of chest CT images, an algorithm can provide a malignancy risk score for lung adenocarcinoma. To this end, the EMV-3D-CNN model was also implemented as a web-based platform for user-friendly access. Figure 4 shows a screenshot of the application that is available at https://seeyourlung.com.cn. To estimate the probability of lung cancer, users need to upload a series of chest CT images in DICOM format. By manually typing the coordinates of the center point (i.e., the *X, Y*, and *Z* coordinates) of a specific pulmonary nodule, our algorithm can give the probability of malignancy (step 1), the probability of invasiveness (this is step 2 if step 1 is passed), and the probabilities of the three risk grades (this is step 3 if step 2 is passed). For a more detailed description of this web-based platform, we refer to Supplementary Note 3 or the user guidelines for the platform that can be downloaded from the website. To further help users to understand and use the platform, we also provide a tutorial video in the online supplementary. Readers can directly visit the video by clicking https://github.com/zhoujing89/EMV-3D-CNN.

## Discussion

Currently, for early-stage lung cancer study, the radiographic diagnosis of pulmonary nodules and the appropriate selection of the surgical procedure remain two popular topics for researchers. Among all lung cancer types, lung adenocarcinoma accounts for more than 75% of early-stage lung cancer^48^. Therefore, it is of significant importance to develop an accurate diagnostic model for pulmonary nodule classification. However, most current automatic diagnosis models focus on identifying benign and malignant pulmonary nodules that are detected in screening or incidentally^35–37^. Both of them are with very low malignant rate and more likely work for less suspicious nodules. Meanwhile, no techniques have been developed for risk stratification of invasive lung adenocarcinoma from the perspective of surgical evaluation. Such an AI tool can potentially work for suspicious nodules that are candidates for surgery. In this study, we developed an EMV-3D-CNN model for lung adenocarcinoma risk stratification on thin-slice CT scans. We trained and evaluated the model using data collected from three medical centers that were all undergoing surgeries. That means our tool more likely work for highly suspicious nodules that will undergo a surgery. The proposed model was evaluated on three tasks: (1) detecting benign and malignant pulmonary nodules; (2) identifying Pre-IA and IAC; (3) and classifying the risk stratification level (i.e., Grades 1, 2, 3) for invasive lung adenocarcinoma. Our model achieved excellent performance for all three tasks. Furthermore, in an observational comparison study with six radiologists, our model also achieved an equivalent or even higher performance compared with senior doctors having over 10 years of clinical experience. Finally, for user-friendly access, we developed an online platform embedded with the proposed EMV-3D-CNN model (https://seeyourlung.com.cn). This platform can assist clinicians in lung nodule diagnosis and surgical procedure selection.

Detecting benign and malignant pulmonary nodules using chest CT images is one of the most popular topics in deep learning. From Table 1, we can see that compared with benign nodules, malignant nodules showed larger average nodule diameter. However, this correlation is not that simple as a linear relation (see Supplementary Fig. 4). As such, size is not always a discriminator for benign vs malignant discrimination. Previous studies have mainly used public datasets such as LIDC-IDRI (or LUNA2016) and NLST^23,24,27,49^. All the subjects from the two datasets were from the United States. However, there are some distribution differences in the epidemiologic features of pulmonary nodules between the Chinese and American populations^50^. For example, nodules containing ground-glass opacity (GGO) components in both datasets are very rare, while GGO accounts for a large proportion of the Chinese population^51^. Therefore, a direct application of the previous models may lead to biased results. In this study, we collected CT images from three medical institutions in China. The proposed EMV-3D-CNN model has been verified and achieved satisfactory accuracy and stability. From Table 1, we can see that the nodules with different radiomic characteristics were relatively evenly distributed in the training set. This leads to a better prediction performance in the four nodule types (i.e., pure ground-glass, heterogeneous ground-glass, part-solid, and pure solid nodules). In the comparison study with six doctors (see the design of the observational study in the Methods section), the EMV-3D-CNN model achieved equivalent or slightly higher performance compared with the senior doctors and much higher performance than junior doctors. Since early detection of small pulmonary nodules is less sensitive to liquid biopsy, puncture diagnosis, and other detection methods^52,53^, it is of great clinical value to detect benign and malignant nodules by CT images. Although biopsy is also an important and accurate method for the preoperative diagnosis, it is an invasive operation and examination. Besides, for about 16.7–29.8% of the patients, clinicians are unable to obtain tumor tissue biopsy or evaluable tissue biopsy (were non-diagnostic). Previous studies revealed that tumors with less than 15 mm and non-solid imaging feature can lead to a decrease of accuracy rate^54,55^. In China, due to the large number of patients, performing biopsy on all suspicious nodules will inevitably overcrowd medical resources. Therefore, our tool can effectively screen out patients with a higher possibility of malignancy. For those patients with a relatively high score in Task 1, AI diagnosis may replace biopsy to a certain extent. Nevertheless, identifying benign and malignant nodules only addresses the question of whether patients need further surgical intervention. To select the suitable surgical treatment, thoracic surgeons need to evaluate and predict the malignant risk of the nodules.

The second task of our study was to distinguish between Pre-IA and IAC. Radiologists and thoracic surgeons usually perform a visual evaluation by measuring the proportion of GGO or solid components on 2D CT images. Therefore, individual differences in judgement and bias are inevitable. For example, in defining GGO and solid components, personal preferences will cause controversy in clinical practice. However, deep learning models usually have good stability in this regard. In previous studies, the AUC values for classifying Pre-IA and IAC were between 83 and 92%^10,29,40,41^. In this study, we achieved an AUC value of 92.93%, which is slightly higher than previous ones. A recent paper has further classified AAH, AIS, and MIA in pre-invasive lesions using CT images^56,57^. However, since there is no significant difference in the treatment strategy and prognosis of these three types of pre-invasive nodules in clinical practice, we did not identify these three Pre-IA subtypes in this study.

Recently, as additional insights into early-stage lung adenocarcinoma, the surgical treatments for early-stage lung adenocarcinoma have gradually changed. With the release of the results for two clinical trials, JCOG0802^58^ and CALGB140503^59^, sublobar resection has been established as a surgical treatment of early-stage lung cancer. However, many studies have found that IAC with micropapillary or solid pattern results in a high relapse rate^44,60,61^. For patients with predominant and high-grade patterns (solid, micropapillary, or complex gland), lobectomy may still be the best suitable surgical treatment. Due to the different histologic subtypes of adenocarcinoma associated with different prognoses, the latest IASLC grading system classifies pulmonary IAC into three grades: Grade 1 (well differentiated), Grade 2 (moderately differentiated), and Grade 3 (poorly differentiated)^7^. Hence, if the pathological grades of IAC can be accurately predicted using preoperative CT images, it will effectively assist thoracic surgeons in choosing the appropriate surgical treatment. In the current study, we used a deep learning-based model to predict the pathological grade of early-stage IAC for the first time. Our model achieved an accuracy of 77.6%, which is much higher than that obtained by senior radiologists in the observational study, demonstrating the significant advantage of AI-based methods in predicting the pathological subtypes of IAC. Given the large training size (average 3500 chest CT per year) and train years (average 13 years) of senior radiologists, one of the advantages of the AI-model is its efficiency. Our model, which was trained on only 843 nodules, has surpassed experienced clinical doctors in tumor pathological grading. This indicates that AI model can extract details of tumor more comprehensively and effectively than the naked eye, and are superior to doctors in identifying pathological subtypes. It is well known that there are distinct radiographic features in the differentiation of benign, malignant, and pre-invasive nodules, which can help clinicians identify these nodules. For example, MIA shows a ground-glass predominant nodule 3 cm or smaller with a solid component that should appear 0.5 cm or small^6^. However, there is a lack of typical CT imaging features among histological grades in invasive pulmonary adenocarcinoma. In this regard, it disfavors thoracic surgeons in evaluating the risk of nodules using 2D CT images. Although in some previous studies, pathologists could identify the high-grade patterns of lung adenocarcinoma by intraoperative frozen sections^44,62^, the detection consistency among different pathologists was unsatisfactory, with an overall accuracy of approximately 74%. Meanwhile, relying solely on the intraoperative frozen results to select the surgical treatment of invasive lung adenocarcinoma may affect the implementation of segmentectomy and the extent of lymph node dissection. Besides, given that the small amount of tissue examined with pre-surgical biopsy, a poor agreement was observed between post-surgical histology and pre-surgical biopsy for pathological grade. In our study, we developed an AI-based classification algorithm for invasive pulmonary adenocarcinoma using CT images (Task 3). The classification is based on the latest grading system established by the IASLC pathology panel. Our model achieved an accuracy of 88.2%, 78.9%, and 88.2% for well (Grade1), moderately (Grade2), and poorly (Grade3) differentiated adenocarcinoma, respectively. In terms of risk stratification of invasive lung adenocarcinoma, our results are superior and slightly better than those achieved by doctors and intraoperative frozen pathology, respectively. Thus, it can effectively assist thoracic surgeons in selecting surgical treatments because these prediction results are based on preoperative CT scans.

To further validate the external validity of the proposed model, we included a completely independent external dataset collected from Beijing LIANGXIANG Hospital. It contained 194 pulmonary nodules, covering 132 patients (47 men, 85 women, mean age of 57.9 ± 11.4 years), who have undergone surgeries between Jan 2016 and Dec 2021. We then apply the proposed EMV-3D-CNN model and the determined cutoff values on this dataset. The detailed performance results are reported in Supplementary Table 4. For task 1, with the determined cutoff value of 0.747, the overall accuracy of distinguishing between benign and malignant is 89.7%. For task 2, with the determined cutoff value of 0.46, the overall accuracy of classifying between pre-invasive tumor and invasive tumor is 83.8%. For task 3, the overall accuracy for distinguishing among the three different grades is 70.4%. From these results, we can conclude that the cutoffs that were determined based on the current validation set can also generalize to other datasets, since the performance on this external dataset is relatively stable. For other more detailed results, we refer to Supplementary Table 4.

Despite the promising results, our study has the following limitations. First, the proposed EMV-3D-CNN model was developed and evaluated using data collected only from three cooperative hospitals, and the number of samples was relatively small. To further validate the clinical application value of the model, more samples should be collected and used to perform comprehensive external validation. Second, the proposed model was developed based on thin-slice CT images, which limits the usage of CT images with fewer slices (e.g., slice thickness is larger than 5 mm). Although a previous study indicated that the low resolution of CT images can decrease the performance of deep learning models^43^, developing an algorithm that can combine both high- and low-resolution CT images remains a significant challenge that should be addressed in future research. Finally, both the EMV-3D-CNN model and doctors in the observational study were blinded to other clinical information, which was slightly different from real-world clinical situations. Including clinical information may provide additional diagnostic information to the risk stratification of nodules. Furthermore, previous studies have suggested that aggregating deep learning and radiomic features can help improve the classification accuracy of Pre-IA and IAC^20^. Therefore, there is a need for developing a comprehensive framework that incorporates radiomic features, clinical information, and deep learning to predict the risk stratification of lung cancer.

## Methods

### Data preprocessing and augmentation

This multicenter study was performed in four centers in China. The requirements for informed consent were waived owing to the study’s retrospective nature. The study was conducted in accordance with the Declaration of Helsinki (as revised in 2013) and approved by the ethics committee of Beijing Chao-Yang Hospital (No. 2022-ke-36). The Third Xiangya Hospital, Central South University, Changsha central hospital, Beijing LIANGXIANG hospital and Renmin University of China were informed and agreed with this study.

**Fig. 5:**
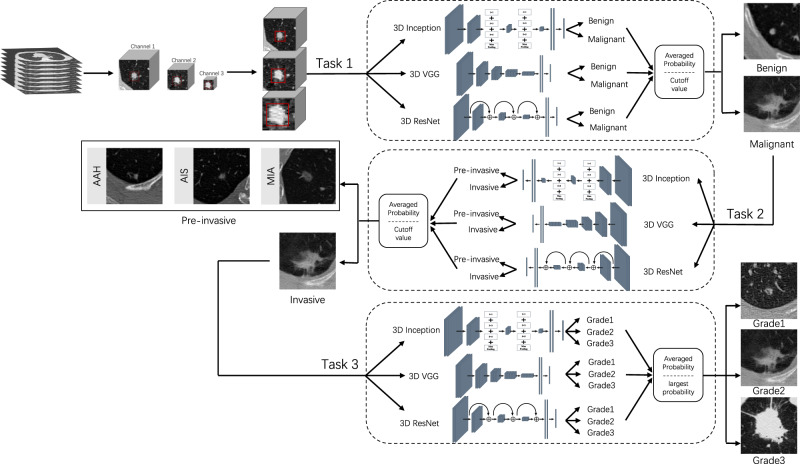
The workflow of model development. Task 1 classifies benign and malignant lung nodules. Task 2 identifies pre-invasive and invasive malignant nodules. Task 3 further evaluates the risk stratification of invasive lung adenocarcinoma. It is remarkable that for each task, the input shapes of the three 3D CNN are different with each other. Their detailed input shapes of the three 3D CNN for each task are list in the Supplemental Table 2.

We performed data preprocessing on the CT images to apply the proposed EMV-3D-CNN model to our three tasks (e.g., Fig. 5). Specifically, to eliminate the variations in image resolution and slice thickness in different CT scans, the CT images were interpolated to a fixed voxel spacing of 0.625 mm × 0.625 mm × 0.625 mm. Then, the CT values of each scan were normalized to [0,1] by applying a window range of [-1400HU, 400HU] using the min-max normalization method. Next, inspired by the studies of Liu et al. and Liu et al.^63,64^, we developed a multi-view strategy to capture the detailed characteristics of nodules with different sizes. The input for each 3D CNN model in all the three tasks had to be 3D patches with three different views. In this study, for each pulmonary nodule, we considered cropping the 3D patches with five different views based on the coordinate values of the center point. The 3D patches had voxel sizes of 100 × 100 × 100, 80 × 80 × 80, 60 × 60 × 60, 40 × 40 × 40, and 20 × 20 × 20. For simplicity, a 3D patch with a voxel size of 100 × 100 × 100 is written as 3D-100, and so on for others. To this end, a total of ${C}_{5}^{3}=10$ combinations of three views need to be examined for each 3D CNN model in each task. To determine the best view size combination, we conducted several experiments based on the training and validation cohorts. Then, the view size combination that maximized the accuracy of the validation set was chosen as the input of the corresponding model. Supplementary Table 5 shows the three tasks' final view size combinations for each 3D CNN model. Lastly, to adapt the input for the subsequent modeling process, we unified the 3D patches with different views into a predefined feeding voxel size using a spline interpolation algorithm (see the last column in Supplementary Table 5).

Second, we employed a series of data augmentation techniques to further increase the sample size in the training cohort. These techniques included: (1) rotating and flipping the 3D patches on the *X, Y*, and *Z* axis; (2) rotating the 3D patches by 36 degrees from 0 to 360 degrees; (3) random shifting; and (4) nodules with a diameter of less than or equal to 1.5 centimeters were randomly amplified by 1.5 times. To this end, the sample size of the training cohort was expanded to 28,782 pulmonary nodules. Specifically, the actual numbers of pulmonary nodules used for training were 13,635, 10,158, and 4989 for Tasks 1, 2, and 3, respectively. Supplementary Fig. 1 shows a flowchart of data preprocessing and augmentation.

### Pathological evaluation

The pathological sections used in this study were reviewed by four experienced senior pathologists. Pathologic diagnosis was based on the 5th edition WHO classification of lung tumors. IAC was further divided into well (Grade 1), moderately (Grade 2), and poorly (Grade 3) differentiated adenocarcinoma based on the grading system proposed by the IASLC pathology panel. Following Moreira et al.^7^, Grade 1 is well-differentiated adenocarcinoma: lepidic predominant tumors with no or less than 20% of high-grade patterns (solid, micropapillary, and/or complex glandular patterns); Grade 2 is moderated differentiated adenocarcinoma: acinar or papillary predominant tumors with no or less than 20% of high-grade patterns; and Grade 3 is poorly differentiated adenocarcinoma: any tumor with 20% or more of high-grade patterns.

### Model development

Figure 5 shows the flowchart of the proposed EMV-3D-CNN model. To build the proposed model, multi-view 3D patches preprocessed from original CT images were fed into the model as input. Our approach aims to accomplish three key tasks: detection of benign and malignant lung tumors (Task 1), classification of Pre-IA and IAC (Task 2), and risk stratification (i.e., Grades 1, 2, and 3) of invasive lung tumors (Task 3)^7^. For each task, we first independently trained 3D Inception, 3D VGG, and 3D ResNet models on the training set with pathological types as targeted labels. After training, each 3D CNN model can give a predicted probability of each category for each pulmonary nodule in the validation dataset. We then calculate an averaged probability of each category for each nodule. For Task 1 and Task 2, a cut-off value determined using the Youden index was adopted to obtain the final predicted label. For Task 3, the category corresponding to the maximum averaged prediction probability was used as the final predicted label. In brief, we introduce the architectures of the three 3D CNN models in the next paragraph. For illustration purpose, the details of the proposed models for Task 1 are shown in Supplementary Tables 6–8. It should be noted that model structures for Task 2 and Task 3 can be obtained by simply changing the input shapes, and we have eliminated them for space saving.

In deep learning, 3D CNNs are used for 3D feature extraction. The key point is to replace the 2D convolution and pooling operations in the traditional 2D CNN with 3D convolution and pooling operations. This is to accommodate the multi-view 3D input patches (i.e., 40 × 40 × 40). For the 3D inception model, there are a total of 145 layers, including nine inception modules^65^. Each inception module comprises six convolution layers, six batch normalization layers, and two pooling layers. Inside each inception module, we concatenate all the layers in the channel dimension at the last step. Before the classification layer, we adopt a dropout layer with an inactivation rate of 0.4. The specific size and number of convolutional kernels are displayed in Supplementary Table 6. The 3D VGG model has 27 layers, including five convolutional blocks^66^. Each convolutional block has either two or three convolutional layers, one pooling layer, and one batch normalization layer. Before the classification layer, we adopt a dropout layer with an inactivation rate of 0.4 and a max pooling layer. The specific size and number of convolutional kernels are displayed in Supplementary Table 7. Lastly, the 3D ResNet model has 87 layers, including eight residual blocks^67^, designed to facilitate the model construction. Each residual block comprises three convolutional layers, five batch normalization layers, and a short connection to combine the input and output in each block. Before the classification layer, we adopt an average pooling layer, a dropout layer with an inactivation rate of 0.4, and a 3D pooling layer. The specific size and number of convolutional kernels are displayed in Supplementary Table 8.

The EMV-3D-CNN model was implemented using Python 3.9 based on TensorFlow 2.8deep learning library and trained on multiple NVIDIA P100 GPUs with 16 GB memory. To obtain a relatively high model performance, we apply both a naïve grid search and a random search strategy for selecting the optimal hyper-parameters. Specifically, we consider the combination of three hyper-parameters, they are the learning rate (i.e., [${10}^{-4},{10}^{-1}$]), optimizer (i.e., Adam, SGD, Adagrad, Adadelta, and Nadam), and learning patience epochs (i.e., [5,10,20]). For each individual model in the three tasks, we validate 50 different sets of hyper parameter combinations. To determine the optimal parameter set, we conducted a total of 20 epochs to train the individual model corresponds to a specific hyperparameter set, and recorded the maximum prediction accuracy. Then the hyperparameter combination with the highest accuracy on the validation is chosen as the final parameter set for each individual model. In summary, except for the 3D Inception and 3D VGG model in task 2, all the other models were trained using the Adam optimization algorithm with an initial learning rate of 0.001. When the prediction accuracy did not improve after every 20 epochs, the learning rate was reduced to half of the previous one. Then we adopt Nadam optimization algorithm with an initial learning rate of 0.0032 for 3D Inception and 0.001 for 3D VGG, respectively in task 2. For all the models, the batch sizes of the training and validation sets were fixed to 30 and 20, respectively. Finally, a total of 200 epochs were conducted for each model. To further accelerate the training process, all of the models were trained using an online GPU platform (https://matpool.com). This enables us to use multiple GPUs to run the models in a parallel manner. For each model, we chose the epoch with the maximum prediction accuracy on the validation as the final evaluated model and release the H5 file in public (see code availability).

### Observational study design

To demonstrate the effectiveness of the proposed method, we conducted an observational study to compare the performance of the EMV-3D-CNN model with that of six doctors on the validation dataset. Six doctors from hospitals (Beijing Chao-Yang Hospital and The Third Xiangya Hospital, Central South University) with 2–15 years of clinical experience were recruited and divided into two groups based on their chest CT interpretation experience: senior (with an average clinical experience of 13 years) and junior groups (with an average clinical experience of 3 years). In each group, three radiologists independently completed the three tasks. For Tasks 1 and 2, we followed the reference standard of diagnostics by ref. ^43^. Specifically, for Task 1, we used scores from 1 to 5 to represent highly suspicious normal/benign to highly suspicious malignant nodules. Similarly, we also used scores 1–5 to describe highly unlikely invasive to highly suspicious invasive nodules for Task 2. Finally, for Task 3, we directly asked the radiologist to choose the grade (i.e., Grades 1, 2, and 3) that the node is most likely to belong to. All the radiologists were blinded to the specific pathological results and other clinical information to determine the malignant degree of pulmonary nodules. Since our algorithm can provide a result in a few seconds, for a fair comparison, we asked each radiologist to provide an answer within 30 s for each pulmonary nodule.

### Statistical analysis

The performance of the proposed EMV-3-CNN model was comprehensively evaluated with seven metrics: AUC, accuracy, sensitivity, specificity, PPV, NPV, and F1 score (${\rm{F}}1=\frac{2\times {Precision}\times {Recall}}{{Precision}+{Recall}}$). To obtain the above metrics, we had to give a predictive probability for each task. To this end, for each task, we first calculated the average predictive probability over the three 3D CNN models. Then, this value was treated as the final predictive probability value for each task. Specifically, for Tasks 1 and 2, the predictive probabilities were then converted into binary results using a threshold determined by the Youden index. The values were 0.747 and 0.46 for Tasks 1 and 2, respectively. Since Task 3 was a multi classification problem, we directly chose the label corresponding to the maximum predictive probability as the predictive result. Additionally, the ROC curve was generated, and the area under it (AUC) was computed using Python. All confidence intervals of AUC values were computed using a bootstrapping method. The Cohen’s kappa values were also computed using Python.

### Reporting summary

Further information on research design is available in the Nature Research Reporting Summary linked to this article.

## Supplementary information

Supplementary information

Supplementary Movie 1

Reporting Summary

## Data Availability

The data are not publicly available due to hospital regulations. However, data requests with aims will be needed to assess the reasonability. After approval from the hospital and the corresponding authors, de-identified CT data will be provided. Requests to access the datasets should be directed to corresponding author.

## Code availability

The code used for preprocessing the CT data, training the models and the parameters of the EMV-3D-CNN model has been made publicly available at Github (https://github.com/zhoujing89/EMV-3D-CNN). We have also packaged our model into an open-access and ready-to-use tool (https://seeyourlung.com.cn/) for the community to test and feedback.
