# Supplementary material for: An ensemble deep learning model for risk stratification of invasive lung adenocarcinoma using thin-slice CT
Source: NPJ Digit Med. 2023 Jul 5;6:119. doi: 10.1038/s41746-023-00866-z (PMC10322969; doi:10.1038/s41746-023-00866-z)
Supplement: Supplementary file 1 — Supplementary information [file 41746_2023_866_MOESM1_ESM.docx]

**Supplementary Information of “An Ensemble Deep Learning Model for Risk** **Stratification of Invasive Lung Adenocarcinoma Using Thin-Slice CT”**

Jing Zhou^#1^, Bin Hu^#2^, Wei Feng^3^, Zhang Zhang^4^, Xiaotong Fu^1^, Handie Shao^1^, Hansheng Wang^5^, Longyu Jin^3^, Siyuan Ai^6^, and Ying Ji^2*^

^1^ Center for Applied Statistics, School of Statistics, Renmin University of China, Beijing, China.

^2^ Department of Thoracic Surgery, Beijing Institute of Respiratory Medicine and Beijing Chao-Yang Hospital, Capital Medical University, Beijing, China.

^3^ Department of Cardiothoracic Surgery, The Third Xiangya Hospital of Central South University, Changsha, China

^4^ Department of Thoracic Surgery, Changsha Central Hospital, Changsha, China.

^5^ Guanghua School of Management, Peking University, Beijing, China.

^6^ Department of Thoracic Surgery, Beijing LIANGXIANG Hospital, Beijing, China.

^#^ Contributed equally.

**Correspondence to:** Ying Ji, MD. Department of Thoracic Surgery, Beijing Institute of Respiratory Medicine and Beijing Chao-Yang Hospital, Capital Medical University, No. 8 GongTiNanLu, Chao-Yang District, Beijing 100020, China.

Email: 15675112499@163.com.

**Supplementary Note 1**

***Inclusion/Exclusion Criteria*:** The registered patients in this study were from three medical centers. The inclusion/exclusion criteria were as follows. Inclusion criteria: (1) age > 18 years; (2) largest diameter of the nodule on the lung window ≤ 30 mm and ≥ 4 mm; (3) all enrolled patients had at least 1 thin-section CT (the layer thickness ≤ 1.5 mm) scan before surgery; (4) the one section thin-layer CT images could be saved as DICOM format data; (5) the diagnosis was made pathological in all enrolled nodules; and (6) the final pathological diagnosis of the nodules was benign or adenocarcinoma (involving AAH, AIS, MIA, and IAC). Exclusion criteria: (1) the pathological diagnosis of the nodule was indeterminate, (ii) the final pathological diagnosis of the nodules was metastatic tumor, and (iii) the patients received neoadjuvant chemotherapy treatment before surgery.

**Supplementary Note 2**

***Detailed Labeling Procedure*:** Nodules were labeled by two experienced thoracic radiologists and one thoracic surgeon in a two-stage process. In the first stage, each radiologist or thoracic surgeon independently annotated the coordinates of the centroid (e.g., *X, Y,* and *Z*) for each pulmonary nodule and its maximum diameter by carefully reading the CT images before surgery. It should be noted that this marking method has been widely used by the famous public LIDC-IDRI dataset ^[1]^. In the second stage, each doctor independently reviewed the labels of other doctors and made some fine adjustments to their own coordinates. The final coordinates (i.e., *X, Y, Z*) of each pulmonary nodule were determined by calculating the median value of all the annotated coordinates.

**Supplementary Note 3**

User Guidelines for the Web-based Platform.

The proposed EMV-3D-CNN model was also implemented as a web-based platform that can be visited by users at (<https://seeyourlung.com.cn>). We will provide detailed instructions of the user manual in this supplementary file.

(Step 1) (Upload DICOM File Folder): The clinicians should first upload a file folder containing all chest CT scans of a patient in the DICOM format. To ensure that the platform can process CT data correctly, the following requirements should be met. 1) There should be no files in NON-DICOM format in the folder, 2) the number of slices should be larger than 100, and 3) only one CT set can be included for one patient (e.g., multiple sets of CT scans of one patient are not allowed). The clinicians can either drag the file folder into the left box or click the “Upload DICOM File Folder” button to complete this step. On average, it takes about 5-10 minutes to upload a folder with 400–500 DICOM files. The uploading time will slightly vary depending on the network bandwidth.

(Step 2) (Mark the Location of the Suspicious Nodule): Once the DICOM files are uploaded successfully, the clinicians can view them in the left box. By default, we display the lung window to view these chest CT images. The clinicians can zoom in or out for the image through the mouse wheel. By sliding the scroll bar below (e.g., which is indicated as the *Z* axis), different slices of CT images can be viewed continuously. To evaluate the risk stratification of a suspicious nodule, first mark the location of the nodule, that is, the specific values of *X, Y,* and *Z* coordinates for the suspicious nodule. To this end, first determine the *Z* coordinate where the maximum diameter of the nodule is located. Second, move the mouse to the center of the nodule. Subsequently, the values of *X, Y,* and *Z* coordinates can be seen in the bottom left corner of the image. The bottom panel of Figure 5 in the manuscript shows an example of CT scan, where we can see the values of *X, Y*, and *Z* coordinates for a suspicious nodule (e.g., marked by a red box) as 180, 276, and 85, respectively. Lastly, these recognized axis values are then entered manually into the calculation box on the right.

(Step 3) (Calculate the Risk Stratification): Once the *X, Y,* and *Z* values are obtained, click the “Start Calculation Step 1” button to start Task 1, which involves calculating the malignant rate of the nodule. It should be noted that this step will take approximately one minute or more because some data are preprocessed before calculating the malignant rate. If the predictive probability is bigger than the cut-off value of 0.747, the nodule is highly suspected as a malignant tumor. Then, Task 2 is automatically triggered, and “Start Calculation Step 2” can be clicked to continue with the prediction of Pre-IA and IA. This step will be executed faster than step 1 due to the availability of preprocessed data from step 1. If the predictive probability is higher than the cut-off value of 0.46, the nodule is highly suspected as an invasive tumor. Subsequently, Task 3 is triggered, and “Start Calculation Step 3” can be clicked to continue with the last task of predicting the degree of differentiation. This completes the calculation of the three tasks. However, if the predictive probability is smaller than the cut-off value in step 1 or step 2, then the calculation will stop and will not go to the next step. If the clinician wants to evaluate multiple nodules, he/she can enter the location of nodules one by one and repeat the above calculation process.

***Remarks.*** The above three steps show how to use the web-based model to evaluate the risk stratification of lung nodules. There are some important remarks to be stated here. First, by uploading the DICOM file folder, the platform does not collect any personal information about patients. In fact, we have desensitized the meta data of DICOM files, and the CT image data will not be stored on the server. Second, when evaluating the risk stratification of the lung nodules, the specific centroid location (i.e., the manually marked *X, Y, Z* values) is very important to the final prediction score. Therefore, it is important to locate the centroid that is the most central position of the nodule. This will be more consistent with the setting of the proposed algorithm. Third, although the proposed EMV-3D-CNN model has achieved good performance based on the trained data, the predicted results are only for reference, NOT for final diagnosis. The final judgment should be made based on the clinical results along with the CT images. Lastly, although we have performed numerous tests on the platform, bugs are inevitable. Users can contact the authors should they encounter any problems. The contact email is [jing.zhou@ruc.edu.cn](mailto:jing.zhou@ruc.edu.cn).

**Supplementary Note 4**

In this part, we provide a detail explanation about how the uploaded data is stored and used. Specifically, the platform contains three functional processes. They are, respectively, 1) upload process; 2) calculation process; and 3) resource cleanup process. We display the three processes independently in the following three charts.


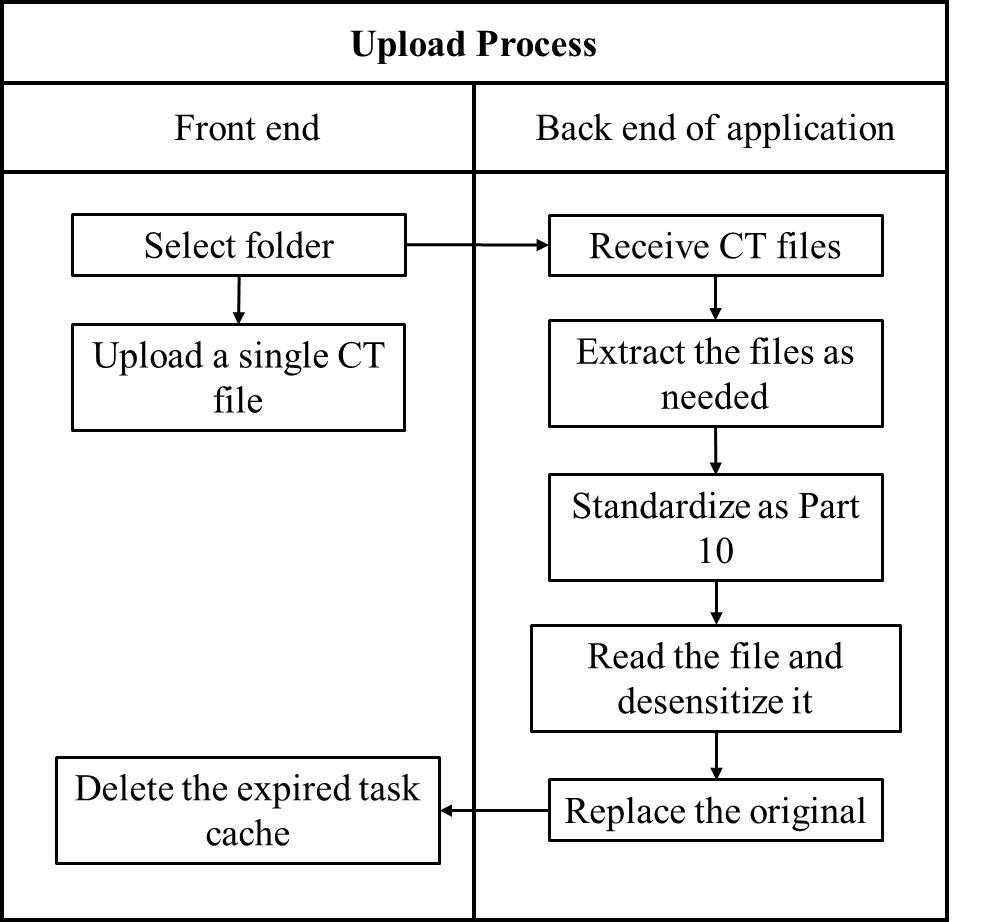


Chart 1 The upload process


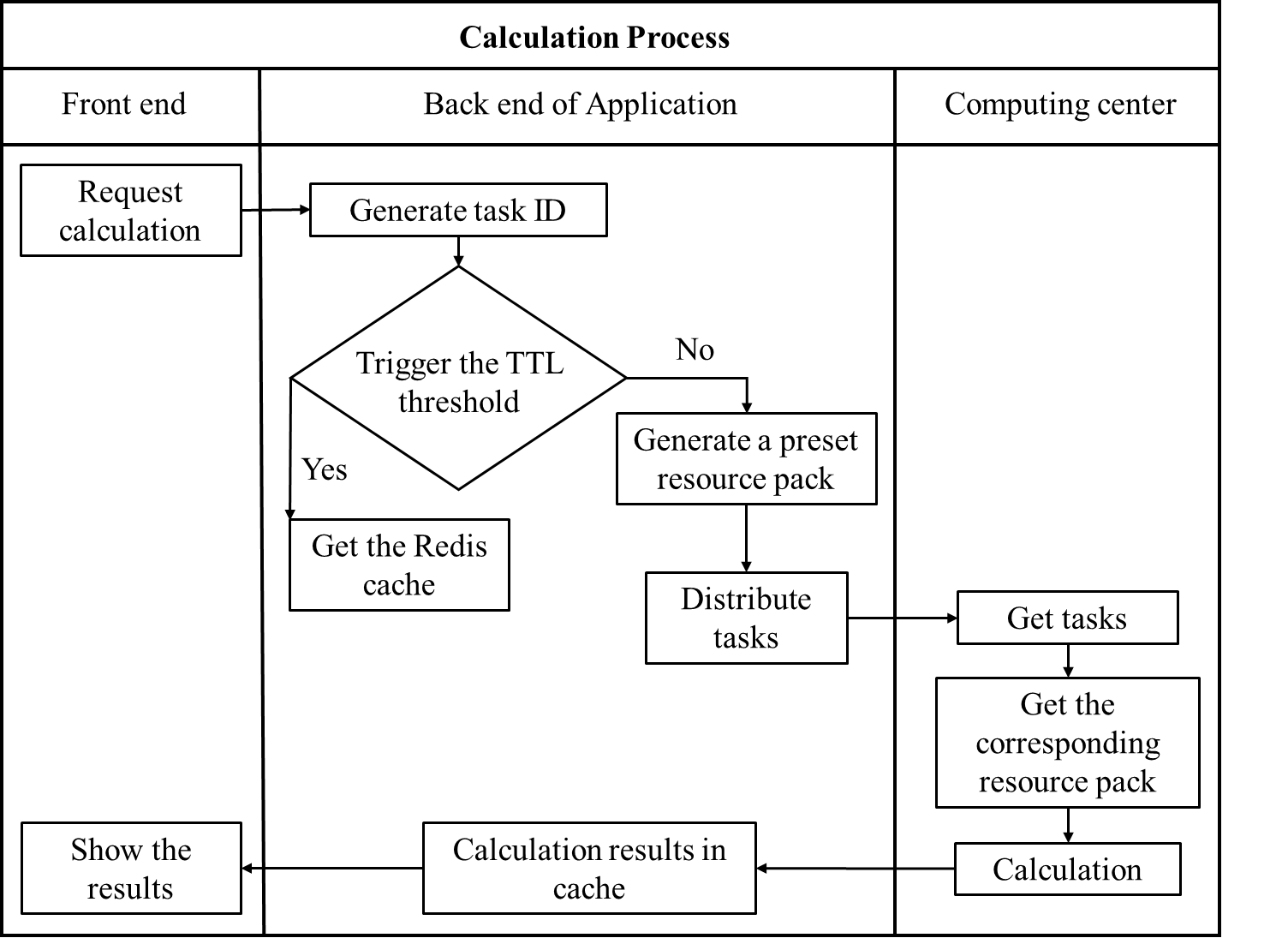


Chart 2 The calculation process


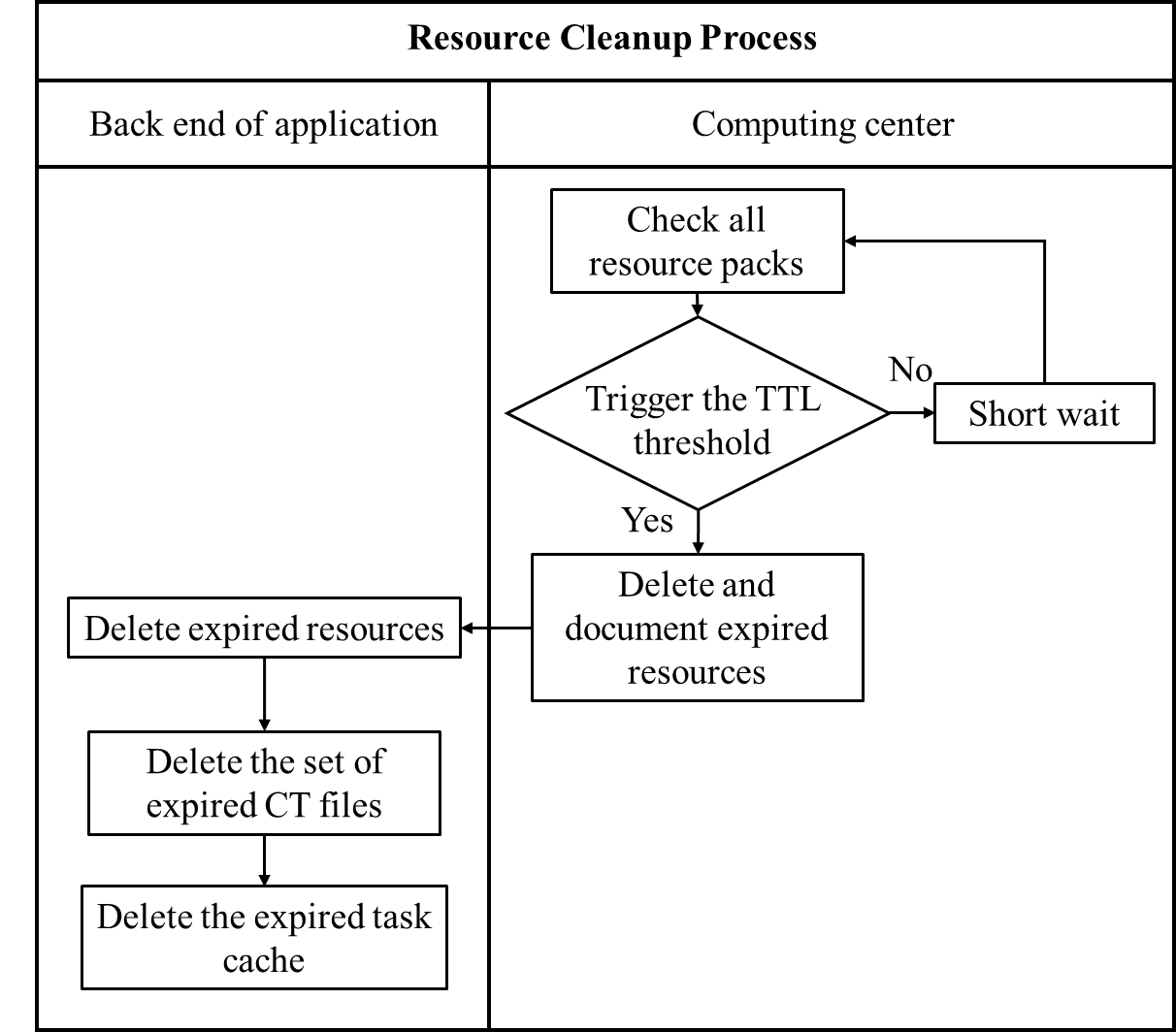


Chart 3 The resource cleanup process

**Supplementary Figure 1**


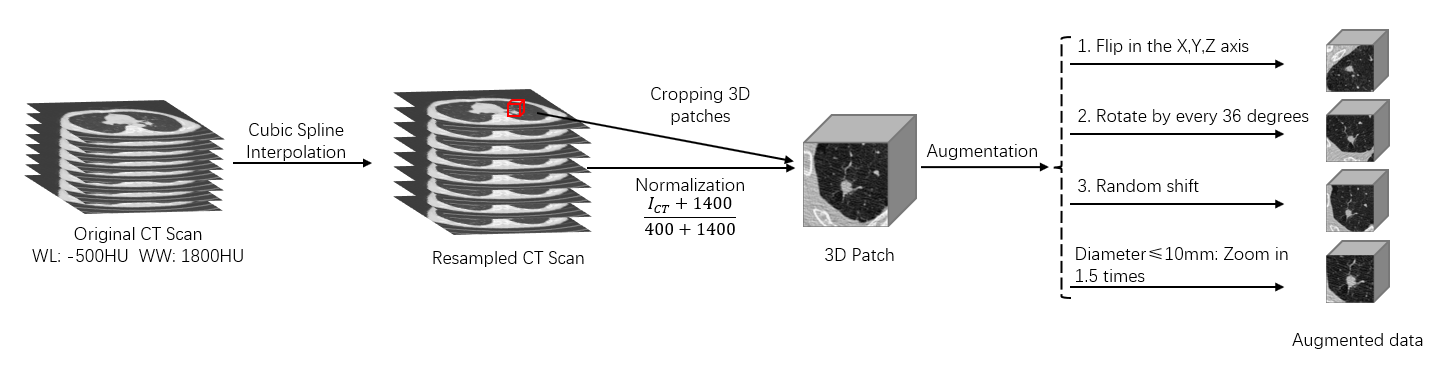


Flowchart of data preprocessing and augmentation.

**Supplementary Figure 2**


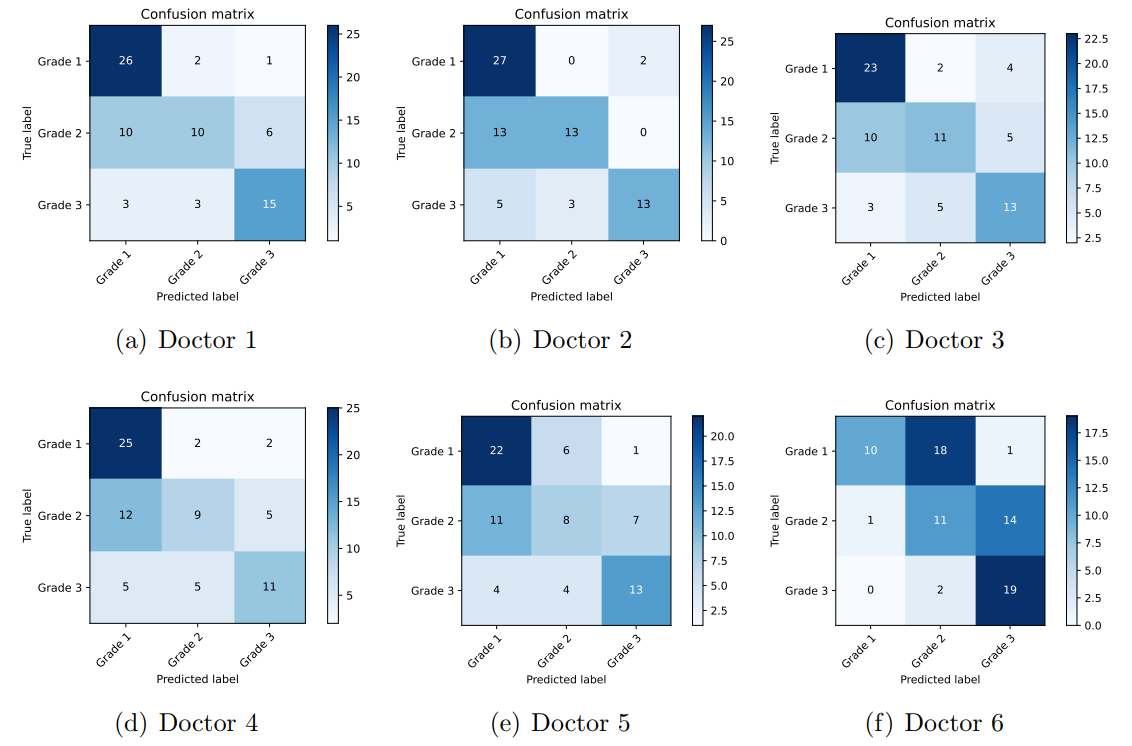


Confusion matrix of identifying the grade risk for the invasive lung nodules in Task 3 for the six radiologists. Doctors 1–3 are from the senior group and Doctors 4–6 are from the junior group.

**Supplementary Figure 3**


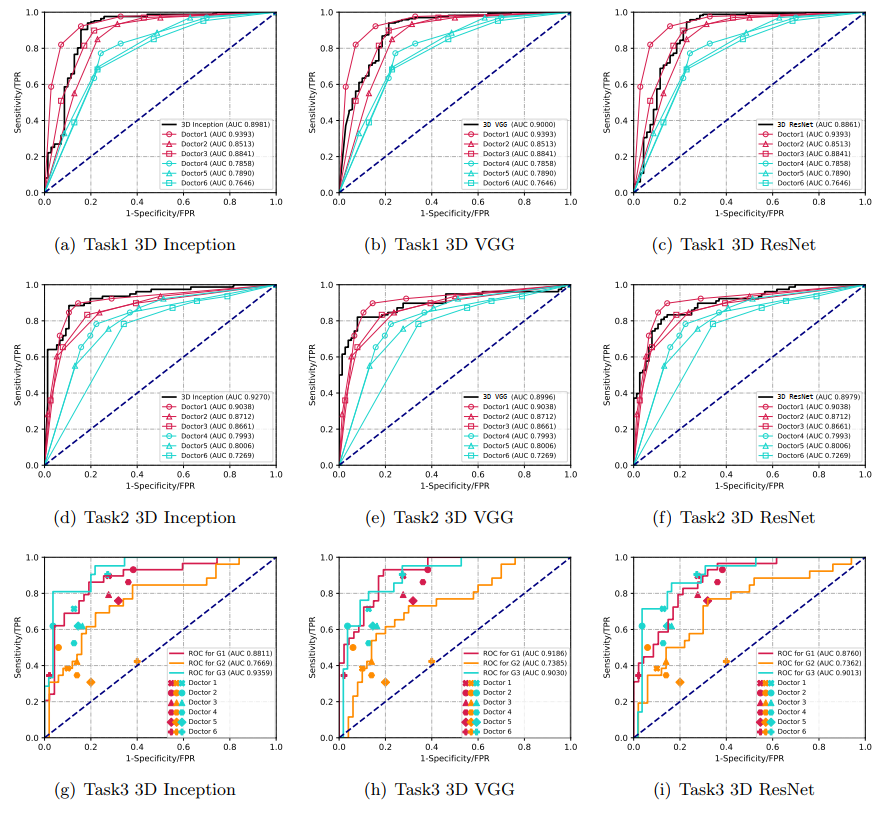


Comparisons of ROC curves and AUC values generated by each individual models in the proposed EMV-3D-CNN model and the six doctors. (a)-(c): The Task 1 ROC curves for three individual models and the six radiologists. (d)-(f): The Task 2 ROC curves for three individual models and the six radiologists. (g)-(i): For the multiclass problem of Task 3, we conduct a pairwise comparison (one class vs. all other classes) and then plot three standard ROC curves for each individual model. For the radiologists’ study, red, yellow, and blue indicate the performance of identifying the nodule risk for Grades 1, 2, and 3, respectively.

**Supplementary Figure 4**


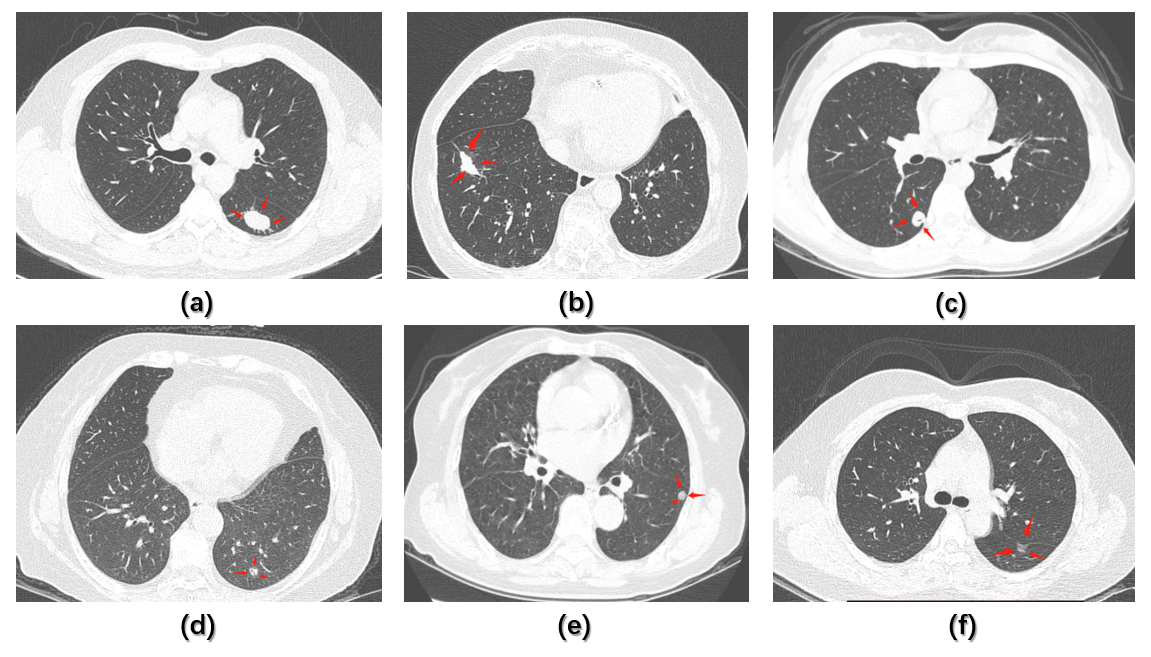
Some particular cases of benign and malignant nodules. The top panel (a)-(c) lists benign nodules with an average size of 20mm, while the bottom panel (d)-(f) lists malignant nodules with an average size of only 8mm.

**Supplementary Table 1**

CT scan characteristics for the three centers in this study.

| Medical Center | Manufacture | Manufacture Model Name | Tube Voltage (kVp) | Slice Thickness (mm) |
| --- | --- | --- | --- | --- |
| Beijing  Chao-Yang  Hospital | GE MEDICAL SYSTEMS  SIEMENS  Philips  NM | iCT 25 | 100/110/120  140/150 | 0.72$\pm0.2$ |
|  |  | BrightSpe |  |  |
|  |  | NeuViz Gl |  |  |
|  |  | Optima CT520 Series |  |  |
|  |  | Revolution Frontier |  |  |
|  |  | SOMATOM Definit |  |  |
|  |  | SOMATOM Force |  |  |
| The Third Xiangya Hospital of Central South University | GE MEDICAL SYSTEMS  SIEMENS  UIH | Revolution CT | 100/110/120 | 0.83$\pm0.1$ |
|  |  | SOMATOM Force |  |  |
|  |  | uCT 710 |  |  |
|  |  | uCT 960+ |  |  |
| Changsha Central Hospital | GE MEDICAL SYSTEMS | BrightSpeed | 120 | 1.25 |
|  |  | LightSpeed VCT |  |  |

**Supplementary Table 2**

Performance comparisons of the EMV-3D-CNN model and each of the individual models for Tasks 1 and 2. PPV = positive predictive value; NPV = negative predictive value

| Evaluation  Index (%) | Task 1 | | | | Task 2 | | | |
| --- | --- | --- | --- | --- | --- | --- | --- | --- |
|  | EMV-3D-CNN | Individual  3D Inception | Individual  3D  VGG | Individual  3D ResNet | EMV-3D-  CNN | Individual  3D Inception | Individual  3D  VGG | Individual  3D ResNet |
| AUC | 91.3 | 89.8 | 90.0 | 88.6 | 92.9 | 92.7 | 89.0 | 89.8 |
| Accuracy | 90.3 | 90.3 | 89.0 | 89.5 | 89.0 | 89.0 | 85.7 | 83.8 |
| Sensitivity | 92.8 | 97.6 | 94.0 | 95.2 | 85.9 | 88.5 | 79.5 | 79.5 |
| Specificity | 84.3 | 72.9 | 77.1 | 75.7 | 92.1 | 89.5 | 92.1 | 88.2 |
| PPV | 93.4 | 89.6 | 90.8 | 90.3 | 91.8 | 89.6 | 91.2 | 87.3 |
| NPV | 83.1 | 92.7 | 84.4 | 86.9 | 86.4 | 88.3 | 81.4 | 80.7 |
| F1 | 93.1 | 93.4 | 92.4 | 92.7 | 88.7 | 89.0 | 84.9 | 83.2 |

**Supplementary Table 3**

Performance comparisons of the EMV-3D-CNN model and each of the individual model for Tasks 3

| Evaluation  Index | Task 3 | | | |
| --- | --- | --- | --- | --- |
|  | EMV-3D-CNN | Individual  3D Inception | Individual  3D VGG | Individual  3D ResNet |
| Accuracy (%) | 77.6 | 69.74 | 73.68 | 69.74 |

**Supplementary Table 4**

Performance for the completely independent external dataset (i.e., Beijing LIANGXIANG Hospital) of the three tasks.

| Evaluation  Index (%) | Task 1 | Task 2 | Task 3 |
| --- | --- | --- | --- |
| Accuracy | 89.7 | 83.8 | 70.4 |
| AUC | 94.1 | 90.1 | - |
| Sensitivity | 92.3 | 84.5 | - |
| Specificity | 82.7 | 83.1 | - |
| PPV | 93.6 | 83.3 | - |
| NPV | 79.6 | 84.3 | - |
| F1 | 92.9 | 83.9 | - |

**Supplementary Table 5**

View size combination for each 3D CNN model for the three tasks. A view size combination displayed as “80-60-40” implies that the three cropped 3D patches are with voxel sizes of 80 × 80 × 80, 60 × 60 × 60, and 40 × 40 × 40. The unified view size displayed as “60-60-60” implies that all the three cropped 3D patches are unified with a voxel size of 60 × 60 × 60. Other expressions in the last columns shall be interpreted in the same way.

| Task | Model | View Size Combination | Unified View Size |
| --- | --- | --- | --- |
| Task 1 | 3D Inception | 60-40-20 | 40-40-40 |
|  | 3D VGG | 60-40-20 | 40-40-40 |
|  | 3D ResNet | 60-40-20 | 40-40-40 |
| Task 2 | 3D Inception | 60-40-20 | 40-40-40 |
|  | 3D VGG | 80-60-40 | 60-60-60 |
|  | 3D ResNet | 60-40-20 | 40-40-40 |
| Task 3 | 3D Inception | 80-60-40 | 60-60-60 |
|  | 3D VGG | 100-80-60 | 80-80-80 |
|  | 3D ResNet | 60-40-20 | 40-40-40 |

**Supplementary Table 6**

Detailed model structure of 3D Inception (take Task 1 as an example)

| Name | | Kernel Size | Output Shape | Parameter |
| --- | --- | --- | --- | --- |
| Input_1 | |  | (3, 40, 40, 40) | 0 |
| conv3d_BN_pooling3d_1 | | 64, (7,7,7) | (64, 10, 10, 10) | 66,176 |
| conv3d_BN_pooling3d_2 | | 192, (3,3,3) | (192,5,5,5) | 332,736 |
| Inception 1 | conv3d_BN_3 | 64, (1,1,1) | (64,5,5,5) | 12,608 |
|  | conv3d_BN_4 | 96, (1,1,1) | (96,5,5,5) | 18,912 |
|  | conv3d_BN_5 | 128, (3,3,3) | (128,5,5,5) | 332,416 |
|  | conv3d_BN_6 | 16, (1,1,1) | (16,5,5,5) | 3,152 |
|  | conv3d_BN_7 | 32, (5,5,5) | (32,5,5,5) | 64,160 |
|  | pooling3d_3 |  | (192,5,5,5) | 0 |
|  | conv3d_BN_8 | 32, (1,1,1) | (32,5,5,5) | 6,304 |
|  | concatenate_1 |  | (256,5,5,5) | 0 |
|  | pooling3d_4 |  | (256,3,3,3) | 0 |
| Inception 2 | |  | (256,2,2,2) | 450,864 |
| Inception 3 | |  | (256,1,1,1) | 450,864 |
| Inception 4 | |  | (256,1,1,1) | 450,864 |
| Inception 5 | |  | (256,1,1,1) | 450,864 |
| Inception 6 | |  | (256,1,1,1) | 450,864 |
| Inception 7 | |  | (256,1,1,1) | 450,864 |
| Inception 8 | |  | (256,1,1,1) | 450,864 |
| Inception 9 | |  | (256,1,1,1) | 450,864 |
| Dropout_1 | |  | (256,1,1,1) | 0 |
| Flatten_dense_1 | |  | (2,) | 514 |
| Total parameter | | 4,443,890 | | |

**Supplementary Table 7**

Detailed model structure of 3D VGG (take Task 1 as an example)

| Name | | Kernel Size | Output Shape | Parameter |
| --- | --- | --- | --- | --- |
| Input_1 | |  | (3, 40, 40, 40) | 0 |
| Conv Block 1 | BN_1 |  | (3, 40, 40, 40) | 12 |
|  | Conv3d_1_2 | 64, (3,3,3) | (64, 40, 40, 40) | 115,904 |
|  | Pooling3d_1 |  | (64, 20, 20, 20) | 0 |
| Conv Block 2 | BN_2 |  | (64, 20, 20, 20) | 256 |
|  | Conv3d_3_4 | 128, (3,3,3) | (128, 20, 20, 20) | 663,808 |
|  | Pooling3d_2 |  | (128, 10, 10, 10) | 0 |
| Conv Block 3 | BN_3 |  | (128, 10, 10, 10) | 512 |
|  | Conv3d_5_6_7 | 256, (3,3,3) | (256, 10, 10, 10) | 4,424,448 |
|  | Pooling3d_3 |  | (256, 10, 10, 10) | 0 |
| Conv Block 4 | BN_4 |  | (256, 5, 5, 5) | 1,024 |
|  | Conv3d_8_9_10 | 512, (3,3,3) | (512, 5, 5, 5) | 17,696,256 |
|  | Pooling3d_4 |  | (512, 2, 2, 2) | 0 |
| Conv Block 5 | BN_5 |  | (512, 2, 2, 2) | 2,048 |
|  | Conv3d_11_12_13 | 512, (3,3,3) | (512, 2, 2, 2) | 21,235,200 |
|  | Pooling3d_5 |  | (512, 1, 1, 1) | 0 |
| Dropout_1 | |  | (512, 1, 1, 1) | 0 |
| Pooling3d_1 | |  | (512,) | 0 |
| Dense_1 | |  | (2,) | 1,026 |
| Total parameter | | 44,140,494 | | |

**Supplementary Table 8**

Detailed model structure of 3D ResNet (take Task 1 as an example)

|  |  | Kernel Size | Output Shape | Parameter |
| --- | --- | --- | --- | --- |
| Input_1 | |  | (3, 40, 40, 40) | 0 |
| Conv3d_pooling3d_1 | | 64, (7,7,7) | (64, 10, 10, 10) | 65,920 |
| Res Block 1 | BN_1 |  | (64, 10, 10, 10) | 256 |
|  | conv3d_BN_2_3 | 64, (3,3,3) | (64, 10, 10, 10) | 221,824 |
|  | BN_2 |  | (64, 10, 10, 10) | 256 |
|  | conv3d_BN_4 | 64, (1,1,1) | (64, 10, 10, 10) | 4,416 |
|  | Activation_1 |  | (64, 10, 10, 10) | 0 |
| Res Block 2 | |  | (64, 10, 10, 10) | 226,752 |
| Res Block 3 | BN_5 |  | (64, 10, 10, 10) | 256 |
|  | conv3d_BN_8_9 | 128, (3,3,3) | (128, 10, 10, 10) | 664,832 |
|  | BN_6 |  | (64, 10, 10, 10) | 256 |
|  | conv3d_BN_10 | 128, (1,1,1) | (128, 10, 10, 10) | 8,832 |
|  | Activation_3 |  | (128, 10, 10, 10) | 0 |
| Res Block 4 | |  | (128, 10, 10, 10) | 904,064 |
| Res Block 5 | BN_9 |  | (128, 10, 10, 10) | 512 |
|  | conv3d_BN_14_15 | 256, (3,3,3) | (256, 10, 10, 10) | 2,656,768 |
|  | BN_10 |  | (128, 10, 10, 10) | 512 |
|  | conv3d_BN_16 | 256, (1,1,1) | (256, 10, 10, 10) | 34,048 |
|  | Activation_5 |  | (256, 10, 10, 10) | 0 |
| Res Block 6 | |  | (256, 10, 10, 10) | 3,610,368 |
| Res Block 7 | BN_13 |  | (256, 10, 10, 10) | 1,024 |
|  | conv3d_BN_20_21 | 512, (3,3,3) | (512, 5, 5, 5) | 10,621,952 |
|  | BN_14 |  | (256, 10, 10, 10) | 1,024 |
|  | conv3d_BN_22 | 512, (1,1,1) | (512, 5, 5, 5) | 133,632 |
|  | Activation_7 |  | (512, 5, 5, 5) | 0 |
| Res Block 8 | |  | (512, 5, 5, 5) | 14,429,696 |
| Average_pooling3d_1 | |  | (512, 5, 5, 5) | 0 |
| Dropout_1 | |  | (512, 5, 5, 5) | 0 |
| Global_average_pooling3d_1 | |  | (512,) | 0 |
| Dense_1 | |  | (2,) | 1,026 |
| Total parameters | | 33,588,226 | | |

**Supplementary References**

[1] A. Setio, A. Traverso, and C. Jacobs. Validation, comparison, and combination of

algorithms for automatic detection of pulmonary nodules in computed tomography

images: the luna16 challenge. Medical Image Analysis, 42:1–13, 2016.
