# Supplementary material for: An ensemble deep learning model for risk stratification of invasive lung adenocarcinoma using thin-slice CT
Source: NPJ Digit Med. 2023 Jul 5;6:119. doi: 10.1038/s41746-023-00866-z (PMC10322969; doi:10.1038/s41746-023-00866-z)
Supplement: Supplementary file 3 — Reporting Summary [file 41746_2023_866_MOESM3_ESM.pdf]

nature portfolio

Corresponding author(s): Ying Ji
Last updated by author(s): Apr 16, 2023



# Reporting Summary

Nature Portfolio wishes to improve the reproducibility of the work that we publish. This form provides structure for consistency and transparency in reporting. For further information on Nature Portfolio policies, see our Editorial Policies and the Editorial Policy Checklist.

## Statistics

For all statistical analyses, confirm that the following items are present in the figure legend, table legend, main text, or Methods section.

| n/a | Confirmed | |
|---|---|---|
| ☐ | ☒ | The exact sample size (*n*) for each experimental group/condition, given as a discrete number and unit of measurement |
| ☐ | ☒ | A statement on whether measurements were taken from distinct samples or whether the same sample was measured repeatedly |
| ☐ | ☒ | The statistical test(s) used AND whether they are one- or two-sided<br>*Only common tests should be described solely by name; describe more complex techniques in the Methods section.* |
| ☒ | ☐ | A description of all covariates tested |
| ☒ | ☐ | A description of any assumptions or corrections, such as tests of normality and adjustment for multiple comparisons |
| ☐ | ☒ | A full description of the statistical parameters including central tendency (e.g. means) or other basic estimates (e.g. regression coefficient) AND variation (e.g. standard deviation) or associated estimates of uncertainty (e.g. confidence intervals) |
| ☒ | ☐ | For null hypothesis testing, the test statistic (e.g. *F*, *t*, *r*) with confidence intervals, effect sizes, degrees of freedom and *P* value noted<br>*Give P values as exact values whenever suitable.* |
| ☒ | ☐ | For Bayesian analysis, information on the choice of priors and Markov chain Monte Carlo settings |
| ☒ | ☐ | For hierarchical and complex designs, identification of the appropriate level for tests and full reporting of outcomes |
| ☒ | ☐ | Estimates of effect sizes (e.g. Cohen's *d*, Pearson's *r*), indicating how they were calculated |

*Our web collection on statistics for biologists contains articles on many of the points above.*

## Software and code

Policy information about availability of computer code

| | |
|---|---|
| Data collection | RadiAnt: a registered DICOM viewer, used to view the CT images and label the location of nodules.<br>Excel: used to record nodule information of patients and observer study results. |
| Data analysis | In performing our analysis, we used the following open source software:<br>Python version 3.9.12<br>Tensorflow version 2.8.0<br>Keras version 2.8.0<br>Matplotlib version 3.5.2<br>Numpy version 1.22.4<br>Pandas version 1.4.2<br>Sklearn version 1.1.1<br>Math version internal block<br>Itertools version internal block<br>Scipy version 1.8.1<br>R version 4.2.0 |

For manuscripts utilizing custom algorithms or software that are central to the research but not yet described in published literature, software must be made available to editors and reviewers. We strongly encourage code deposition in a community repository (e.g. GitHub). See the Nature Portfolio guidelines for submitting code & software for further information.

# Data

Policy information about availability of data

All manuscripts must include a data availability statement. This statement should provide the following information, where applicable:

- Accession codes, unique identifiers, or web links for publicly available datasets
- A description of any restrictions on data availability
- For clinical datasets or third party data, please ensure that the statement adheres to our policy

Additional documents related to this study are available from the corresponding author upon reasonable request. The datasets from all the four medical centers were used under license for the current study and are not publicly available.

# Research involving human participants, their data, or biological material

Policy information about studies with human participants or human data. See also policy information about sex, gender (identity/presentation), and sexual orientation and race, ethnicity and racism.

| | |
|---|---|
| Reporting on sex and gender | This study is a retrospective analysis study using patient CT images. Gender considerations are based on the biological sex of patients enrolled in the hospital. |
| Reporting on race, ethnicity, or other socially relevant groupings | This study doesn't need to classify patients into different categories according to their race, ethnicity or other socially relevant groupings. |
| Population characteristics | For the training set (488 patients covering 843 nodules, M:F=198:290, Age= mean, 58.4; SD, 11.5).<br>For the validation set (139 patients covering 232 nodules, M:F=54:85, Age= mean, 57.7; SD, 11.7)<br>For the completely unseen dataset (132 patients covering 194 nodules, M:F=47:85, Age=mean, 57.9; SD,11.4) |
| Recruitment | Data were collected from patients who have undergone a surgery in the four medical centers between January 2016 and December 2021. |
| Ethics oversight | The study was conducted in accordance with the Declaration of Helsinki (as revised in 2013) and approved by the ethics committee of Beijing Chao-Yang Hospital (No. 2022-ke-36). The Third Xiangya Hospital, Central South University, Changsha central hospital, Beijing Liangxiang Hospital, and Renmin University of China were informed and agreed with this study. The requirements for informed consent were waived owing to the study's retrospective nature. |

Note that full information on the approval of the study protocol must also be provided in the manuscript.

# Field-specific reporting

Please select the one below that is the best fit for your research. If you are not sure, read the appropriate sections before making your selection.

☒ Life sciences ☐ Behavioural & social sciences ☐ Ecological, evolutionary & environmental sciences

For a reference copy of the document with all sections, see nature.com/documents/nr-reporting-summary-flat.pdf

# Life sciences study design

All studies must disclose on these points even when the disclosure is negative.

| | |
|---|---|
| Sample size | To determine the sample size, we have reviewed a number of previous studies that related to our study, and found the sample size was between 300 and 1500 (i.e., the number of nodules). Considering the collection time and financial support, we believe a sample size of around 1000 (i.e, the number of nodules) is enough to train and validate the algorithm. To this end, we collected data from three medical centers to train and validate the model. To evaluate the potential generalization of the proposed model, we need to test the model performance using a completely external validation dataset. To make a comparison, the sample size of the external validation is about the same as the validation dataset that was used to train the model. |
| Data exclusions | For the patients who have undergone surgery between January 2016 and December 2021, we exclude the data with the following criteria: the pathological diagnosis of the nodule was indeterminate, (ii) the final pathological diagnosis of the nodules was metastatic tumor, and (iii) the patients received neoadjuvant chemotherapy treatment before surgery or biopsy. |
| Replication | The developed model was trained and validated on the CT images from three medical centers. Additionally, the model was also tested on a completely unseen external dataset from a fourth medical center. During the testing stage, the model was blinded to the ground truth. We have reported the results on the external dataset in the discussion part and in the supplementary file. The results show that the proposed model can generalize to other dataset. |
| Randomization | For the training and validation set, we randomly selected the patients who have undergone surgery between January 2016 and December 2021 in each medical center. For the reader study, we show the CT images to each of the reader in a random order. |

| | |
|---|---|
| Blinding | The model was blinded to the ground truth during the testing on the completely unseen external dataset. Also, for the reader study, all the readers were blinded to the ground truth of the nodules, as well as the clinical information of the patients. |

# Reporting for specific materials, systems and methods

We require information from authors about some types of materials, experimental systems and methods used in many studies. Here, indicate whether each material, system or method listed is relevant to your study. If you are not sure if a list item applies to your research, read the appropriate section before selecting a response.

## Materials & experimental systems

| n/a | Involved in the study |
|---|---|
| ☒ | ☐ Antibodies |
| ☒ | ☐ Eukaryotic cell lines |
| ☒ | ☐ Palaeontology and archaeology |
| ☒ | ☐ Animals and other organisms |
| ☒ | ☐ Clinical data |
| ☒ | ☐ Dual use research of concern |
| ☒ | ☐ Plants |

## Methods

| n/a | Involved in the study |
|---|---|
| ☒ | ☐ ChIP-seq |
| ☒ | ☐ Flow cytometry |
| ☒ | ☐ MRI-based neuroimaging |
